# Processing Strategies to Enhance the Structural and Functional Properties of Proteins From Sweet Potato By‐Products

**DOI:** 10.1111/1750-3841.71544

**Published:** 2026-10-21

**Authors:** Isabella de Almeida Pina, Ana Clara Troya Raineri Fiocco, Isabela Simões Soares, Carolina Siqueira Franco Picone

**Affiliations:** ^1^ School of Food Engineering Universidade Estadual de Campinas (Unicamp) Campinas Brazil

## Abstract

Sweet potato by‐products are a potential source of proteins and phenolic compounds with valuable functional properties. However, enzymatic browning driven by polyphenol oxidase (PPO) limits their stability and utilization. In this study, processing conditions were designed to achieve controlled partial PPO inactivation using ascorbic acid (AA), sodium metabisulfite (MB), and a combined ascorbic acid and mild heating treatment at 50°C (AB50), compared with a control sample that did not receive any anti‐browning treatment. The effects of these treatments on the structural, emulsifying, and antioxidant properties of sweet potato protein extracts were evaluated. AA treatment altered protein secondary structure and improved emulsifying activity despite reducing protein solubility. MB promoted higher antioxidant activity, associated with increased protein and phenolic contents. FTIR analyses showed changes in functional groups, suggesting protein–phenolic interactions that contributed to the structural modifications observed after treatment. Among the treatments, AB50 showed the best overall performance, enhancing emulsifying properties (EAI 14.86 vs 7.56 m^2^/g from CT), improving emulsion stability, and increasing antioxidant capacity. These effects were associated with protein structural rearrangements, including changes in surface charge and free sulfhydryl content. Overall, the results demonstrate that AB50 can replace sodium metabisulfite while enhancing the functional properties of sweet potato protein extracts. Therefore, controlled processing represents a promising strategy for adding value to sweet potato by‐products as multifunctional food ingredients.

## 1 Introduction

Sweet potato (*Ipomoea batatas* L.) is a widely cultivated root tuber with increasing global production. Sweet potato root contains high amounts of starch and vitamins, and its plant is a natural source of bioactive phenolic compounds (Torres et al. 2021). Currently, sweet potato ranks as the seventh most important food crop worldwide, with annual production close to 93.5 million tons (FAO 2023). Approximately half of this yield is used for starch extraction. However, this process generates effluents containing soluble proteins and other bioactive compounds, leading to the loss of valuable biological resources and contributing significantly to environmental pollution (Hu et al. 2020).

In addition to high starch content, sweet potato contains between 1.9 and 6.4% protein per 100 g of dry matter (de Albuquerque et al. 2019). Sweet potato protein (SPP) has received consideration for its nutritional quality and potential as a functional ingredient in food systems. With a chemical score of 82, SPP presents acceptable nutritive value compared to reference proteins, reflecting its balanced essential amino acid profile, including isoleucine, leucine, lysine, methionine, phenylalanine, threonine, tryptophan, and valine (T. Mu et al. 2017; T. H. Mu et al. 2009). Additionally, SPP is rich in branched‐chain amino acids (BCAAs) and contains sulfur‐containing residues such as cysteine and methionine, which are associated with antioxidant activity and protein cross‐linking (Wan et al. 2024). From a techno‐functional perspective, the isoelectric point (pI) of sweet potato protein, reported between 4.0 and 4.1 for the bulk protein fraction, strongly influences its solubility and aggregation behavior under different pH conditions (T. H. Mu et al. 2009).

Sporamins are the major storage protein of sweet potato, which accounts for approximately 60%–80% of the soluble protein fraction in the tuber and functions not only as a nutrient reservoir but also as a trypsin inhibitor with defensive roles against pests and pathogens (T. Liu et al. 2024). Beyond its biological role in the plant, recent studies have highlighted the functional properties of sporamin, including antioxidant capacity, protease inhibitory activity, and potential contributions to metabolic regulation, which position it as a promising bioactive ingredient for functional foods and nutraceuticals (T. Liu et al. 2024; M. Zhu et al. 2024). However, the sweet potato protein functionality can be compromised by the action of the polyphenol oxidase (PPO) naturally present in sweet potato.

The PPO catalyzes the hydroxylation of monophenols to o‐diphenols, followed by oxidation to o‐quinones. These o‐quinones can then react with amino acids and proteins, forming brown/dark melanin pigments (R. Zhang et al. 2023), compromising the recognized nutritional value of proteins and their functional properties. Several strategies have been employed to inactivate PPO, including thermal treatment, reducing agents, and antioxidants (Sui et al. 2023). Heat treatment is widely used and effectively reduces PPO activity through protein denaturation and structural rearrangements. However, it may also alter the functional properties of other protein fractions, such as solubility and emulsifying capacity (C. Li et al. 2023), compromising the functionality of protein extracts.

Organic acids and inorganic auxiliaries have also been studied for PPO inactivation (Sui et al. 2023; L. Zhou et al. 2020) and expanded application of sweet potato proteins (Fiocco et al. 2025). Ascorbic acid acts as a reducing agent, converting quinones back to phenols and thereby protecting proteins. However, it may also interact directly with amino acid side chains, leading to changes in protein conformation (Luo et al. 2024). Similarly, sodium metabisulfite inhibits PPO by reducing o‐quinones and cleaving disulfide bonds in proteins, which can promote their unfolding (Du et al. 2024). Although widely used in the food industry as an anti‐browning and preservative agent, sodium metabisulfite has been associated with toxicological concerns and potential adverse health effects, which has led to increasing consumer rejection and growing demand for more natural alternatives (Alimohammadi et al. 2021).

Despite these findings, there is still limited understanding of how PPO inactivation treatments influence the structural and functional properties of sweet potato proteins. Such treatments can also interact with and modify other sweet potato proteins, potentially altering conformation, solubility, and functional behavior, as their mechanisms rely on reductive reactions, nucleophilic interactions, or heat‐induced unfolding. Thus, this study aimed to evaluate the effect of processing conditions used for PPO inactivation (sodium metabisulfite, ascorbic acid, and a combined thermal–ascorbic acid treatment) on the emulsifying and antioxidant properties of the protein extracts. The effect of each treatment on the chemical and structural properties of the proteins (zeta potential, hydrodynamic diameter, secondary structure, thermal behavior, protein oxidation) was evaluated and correlated to the functional properties of the protein extracts, such as solubility, antioxidant activity, and emulsifying behavior, bridging the gap between the processing, properties, and performance of the proteins.

## 2 Material and Methods

### 2.1 Materials

The sweet potato (*Ipomoea batatas L*.) tubers were obtained from a local market. L‐ascorbic acid and sodium metabisulfite were acquired from Química Moderna (São Paulo, Brazil). Sunflower oil (Liza/Brazil) was purchased from a local market in Campinas (São Paulo, Brazil). All the other reagents used were of analytical grade.

### 2.2 Methods

#### 2.2.1 Enzymatic Inactivation

Sweet potato roots were peeled with a manual peeler and then cut into approximately 1 cm^3^ cubes. The tuber cubes were immersed in aqueous treatment solutions at a sample‐to‐solution ratio of 1:10 (w/v). The treatments consisted of immersion in 0.80 mM ascorbic acid (AA) for 180 s at 25°C, 0.05 mM sodium metabisulfite (MB) for 180 s at 25°C, and 0.80 mM ascorbic acid at 50°C for 180 s (AB50), combining thermal treatment with an organic acid. The control sample (CT) was maintained at 25°C without additives. Adjustments were made to ensure that all treatments achieved comparable levels of partial PPO inactivation (50%), as verified using a reference methodology for PPO activity (Lim et al. 2019; Manohan and Wai 2012). Each procedure was performed in triplicate.

#### 2.2.2 Protein Extracts

The treated sweet potato cubes were ground with distilled water at a 1:4 (w/v) ratio using a high‐speed blender (550 W; Philips Walita, Brazil) for 1 min. The resulting paste was left to rest for 24 h at 4°C to allow complete starch sedimentation. The supernatant, referred to as starch wastewater, was collected, and its pH was adjusted to 4.1 using 1.0 N HCl. The solution was then kept overnight at 4°C to promote protein precipitation. Then, it was centrifuged at 3000 rpm for 10 minutes, the supernatant was discarded, and the protein precipitate was collected. Finally, the pH of the protein extract was adjusted to 7.0 with 0.1 M NaOH. PPO activity was evaluated to confirm the standardized partial inactivation (Lim et al. 2019), after which the protein extracts were freeze‐dried and stored in a desiccator at 25°C. The protein concentration on each extract was determined by the Kjeldahl method, using a nitrogen‐to‐protein conversion factor of 6.25.

#### 2.2.3 Analyses

##### 2.2.3.1 Zeta Potential and Hydrodynamic Size

The charge density and hydrodynamic diameter of the proteins (0.1% w/v) in ultrapure water were evaluated using a Nano Zetasizer ZS (Malvern Instruments, Herrenberg, Germany) with a detection angle of 173°. The hydrodynamic diameter was measured at pH 7.0. The zeta potential was determined between pH 3.0 and pH 7.0 after pH adjustment with 0.1 M HCl and 0.1 M NaOH. All measurements were performed at 25°C.

##### 2.2.3.2 Total Phenolics

Total phenolics analysis followed the methodology proposed by Fiocco et al. (2025), using the Folin–Ciocalteu reagent. Protein extracts were prepared at 1% (w/v) in distilled water prior to analysis. For the standard curve, gallic acid was used at concentrations of 20, 40, 60, 80, and 100 mg GA/L. Then, 0.25 mL of gallic acid solution or sample was homogenized with 2.75 mL of Folin–Ciocalteu reagent and left to rest in the absence of light for 5 min. After that, 0.25 mL of a 10% sodium carbonate solution was added, and the samples were kept in the dark for 60 min at room temperature. Absorbance was measured at 765 nm using a spectrophotometer.

##### 2.2.3.3 Fourier‐Transform Infrared (FTIR)

Fourier‐transform infrared (FTIR) spectra of the sweet potato protein extracts were obtained using an IRPrestige‐21 FTIR spectrophotometer (Shimadzu, Kyoto, Japan) operated with IRSolution software (version 1.60). Samples were analyzed using the potassium bromide (KBr) pellet method. Approximately 2 mg of sample was mixed with 200 mg of spectroscopic‐grade KBr (1:100, w/w) and thoroughly homogenized using an agate mortar and pestle. The mixture was transferred to a 13‐mm stainless steel die and compressed using a hydraulic press (SSP‐10A, Shimadzu, Kyoto, Japan) connected to a vacuum pump. Pellets were prepared under vacuum by applying a force of 80 kN for 10 min. A pure KBr pellet was used as the background. Spectra were recorded over the range of 400–4000 cm^−^^1^ at a spectral resolution of 4 cm^−^^1^ using 16 scans and Happ–Genzel apodization. The acquired spectra were processed using OriginPro 2024 (OriginLab Corporation, Northampton, MA, USA), including smoothing with a Savitzky–Golay filter (5‐point window) followed by normalization prior to spectral comparison.

##### 2.2.3.4 Circular Dichroism

Circular dichroism (CD) spectra of sweet potato protein extract solutions (0.05% w/v) were recorded using a J‐810 spectropolarimeter (Jasco Corporation, Tokyo, Japan) equipped with a 0.1 mm path length quartz cuvette. Measurements were performed at 25°C over a wavelength range of 190–260 nm, with a 1 nm data interval. The secondary structure content, including α‐helix, β‐sheet, β‐turn, and random coil, was estimated using the *DichroWeb* server (Miles et al. 2022) with the SELCON3 algorithm.

##### 2.2.3.5 Determining Free Sulfhydryl (‐SH) Content

Protein oxidation was determined according to He et al. (2021), based on the total amount of sulfhydryl groups (‐SH) present in the protein extract. To this end, 1 mL of the protein sample (20 mg/mL) was dissolved in 5 mL of tris‐glycine buffer, prepared from 0.086 mol/L Tris, 0.09 mol/L glycine, 4 mmol/L ethylenediaminetetraacetic acid (EDTA), and 8 mol/L urea at pH 8.0. Ellman's reagent (5,5'‐dithiobis‐2‐nitrobenzoic acid) (DTNB, dissolved in tris‐glycine buffer at a concentration of 1 mmol/L) was used. The DTNB reacts with the free sulfhydryl to form an S‐S bond and release TNB, a yellow‐colored compound detected by spectrometry at 412 nm. The amount of sulfhydryl groups (‐SH) was measured according to the following equation:

(1) $$-\mathrm{SH}\left(\mu \mathrm{mol}/g\right)=73.53\times A_{412}\times \frac{D}{C}$$

In which *D* is the dilution factor and *C* is the protein concentration (mg/mL) in the solution.

##### 2.2.3.6 Thermal properties

Thermal analysis of sweet potato protein extracts was conducted using a Differential Scanning Calorimeter (DSC) TA Q2000 (TA Instruments, New Castle, DE, USA). Approximately 9 mg of each protein sample were sealed in aluminum pans and heated from 20°C to 100°C at a constant rate of 10°C/min. An empty, hermetically sealed aluminum pan was used as a reference. The onset temperature (*T_on_*), denaturation temperature (*T_d_*), offset temperature (*T_off_)*, and denaturation enthalpy (*ΔH*) were calculated from the resulting thermograms using the Universal Analysis 2920 software (TA Instruments).

##### 2.2.3.7 Solubility

Protein solubility was analyzed according to Bradford protocol (Bradford 1976). A 0.5% (w/w) protein extract solution was prepared with distilled water and kept on a shaker for 1 h to ensure complete solubilization. The pH of the samples was then adjusted in the range of 2 to 10 with 0.1 M HCl and 0.1 M NaOH, and the solutions were centrifuged at 3000 rpm for 10 min to analyze the soluble protein in the supernatant. The soluble protein content was then determined using the Bradford assay, employing bovine serum albumin (BSA) as the standard protein for the calibration curve and Coomassie Brilliant Blue reagent, with absorbance measured at 595 nm.

##### 2.2.3.8 Antioxidant activity

The antioxidant capacity of the protein extract samples was analyzed using the ABTS method according to Fiocco et al. (2025). For the standard curve, 5 concentrations (100 to 2000 µM) of the trolox standard were used. Briefly, 30 µL of each trolox solution or sample, 1% (w/w), were homogenized with 3.0 mL of the ABTS radical solution and left to rest in the dark for 6 min. The reading was performed at 734 nm. The antioxidant activity was expressed as µM Trolox equivalents per liter of protein extract (µM trolox/L), representing the antioxidant capacity of the recovered extract.

##### 2.2.3.9 Emulsifying capacity and emulsion stability

The emulsifying capacity of the protein extracts was analyzed according to Silva et al. (2022). To this end, sunflower oil and 1% (w/w) protein extract solution were homogenized in a 1:3 (v:v) ratio for 5 min at 8000 rpm using a rotor stator homogenizer (Ultra Turrax T‐10, IKA) at 25°C. Immediately after homogenization, a 50 µL aliquot was withdrawn from the center of the freshly prepared emulsion to ensure representative sampling prior to phase separation and diluted in 5 mL of 0.1% sodium dodecyl sulfate (SDS) solution. Then, the absorbance was measured at 500 nm. The Emulsifying Activity Index (EAI) for each sample was calculated using Equation 2, in which *A* is the absorbance and *c* is the protein sample concentration in g/mL. The sample was also placed in glass test tubes (4 cm initial height (*Hi*), 1.5 cm internal diameter) and observed for approximately 24 h to calculate the creaming index (CI – Equation 3), in which *Hf* is the height of the cream phase layer after 24 h of storage at 25°C.

(2) $$EAI\;\left(m^{2}/g\right)=\frac{2\;\times \;2.303\;\times \;100\;\times \;A}{c\;\times \;0.25\;\times \;10000}\;$$

(3) $$CI\;\left(\%\right)=\frac{Hf}{Hi}\;\times 100$$

Emulsion morphology was evaluated by optical microscopy (JENAVAL 30‐G0020, Carl Zeiss, Göttingen, Germany). Droplet size was quantitatively analyzed using ImageJ software (National Institutes of Health, Bethesda, MD, USA), with at least 200 droplets measured per sample. The Sauter mean diameter (D[3,2]) and Span were calculated according to Equations 4 and 5, respectively.

(4) $$D\left[3,2\right]=\frac{\Sigma d_{i}^{3}}{\Sigma d_{i}^{2}}$$

(5) $$S\;pan=\frac{D_{90}-D_{10}}{D_{50}}$$

In which $d_{i}$ is the droplet diameter measured by ImageJ, and $D_{10}$, $D_{50}$, and $D_{90}$ correspond to the diameters below which 10%, 50%, and 90% of the measured droplets are found, respectively.

#### 2.2.4 Statistical Analysis

All analyses were performed in triplicate. The difference between the means was assessed by ANOVA and Tukey's test, for *p* < 0.05, using the PAST software (Hammer et al. 2001).

## 3 Results and Discussion

Before evaluating the properties of the protein extracts, PPO inhibition was measured to verify the effectiveness of the anti‐browning treatments. AA, MB, and AB50 resulted in PPO inhibition levels of 51.87% ± 2.90%, 53.61% ± 1.61%, and 49.82% ± 2.08%, respectively, with no significant differences among treatments (*p* > 0.05). Therefore, the treatments provided comparable levels of PPO control, ensuring that subsequent differences in protein structure and functionality were associated with the treatment conditions rather than differences in PPO inactivation. This PPO inhibition level was intentionally targeted to balance enzyme control with the preservation of protein functionality.

### 3.1 Zeta Potential and Hydrodynamic Size

To evaluate the surface charge of proteins after enzymatic inactivation treatments, zeta potential was used, since it reflects the electrostatic potential near the particle surface and is commonly associated with colloidal stability and protein aggregation behavior (Z.‐K. Zhao et al. 2018; S. Zhu et al. 2023). Overall, all samples exhibited a similar pH‐dependent trend, with increasingly negative values as pH approached neutrality. At pH 7.0, MB showed more negative values than the control (−36.93 mV and −26.30 mV, respectively), indicating a higher net charge magnitude and greater electrostatic repulsion among particles. Under more negative zeta potential values, colloidal systems are generally expected to be more stable due to a reduced tendency for aggregation. AA‐treated samples also tended to present lower zeta potential values than the control, whereas this pattern was less evident for AB50 (−36.33 mV and −27.73 mV, respectively), possibly due to reduced ascorbic acid effectiveness after blanching.

This behavior was reflected in the hydrodynamic diameter measurements (Figure 1), in which the control sample exhibited the largest diameter at pH 7.0 (237.77 nm), indicating a greater aggregation tendency. In contrast, the MB treatment showed a marked reduction in hydrodynamic diameter (∼151 nm). Sodium metabisulfite has been reported to cleave disulfide bonds and alter protein conformation (Du et al. 2023, Du et al. 2024), which may increase the exposure of ionizable groups and the surface charge density at pH 7. This could explain the higher electrostatic repulsion and smaller particle size observed in MB‐treated samples.

**FIGURE 1:**
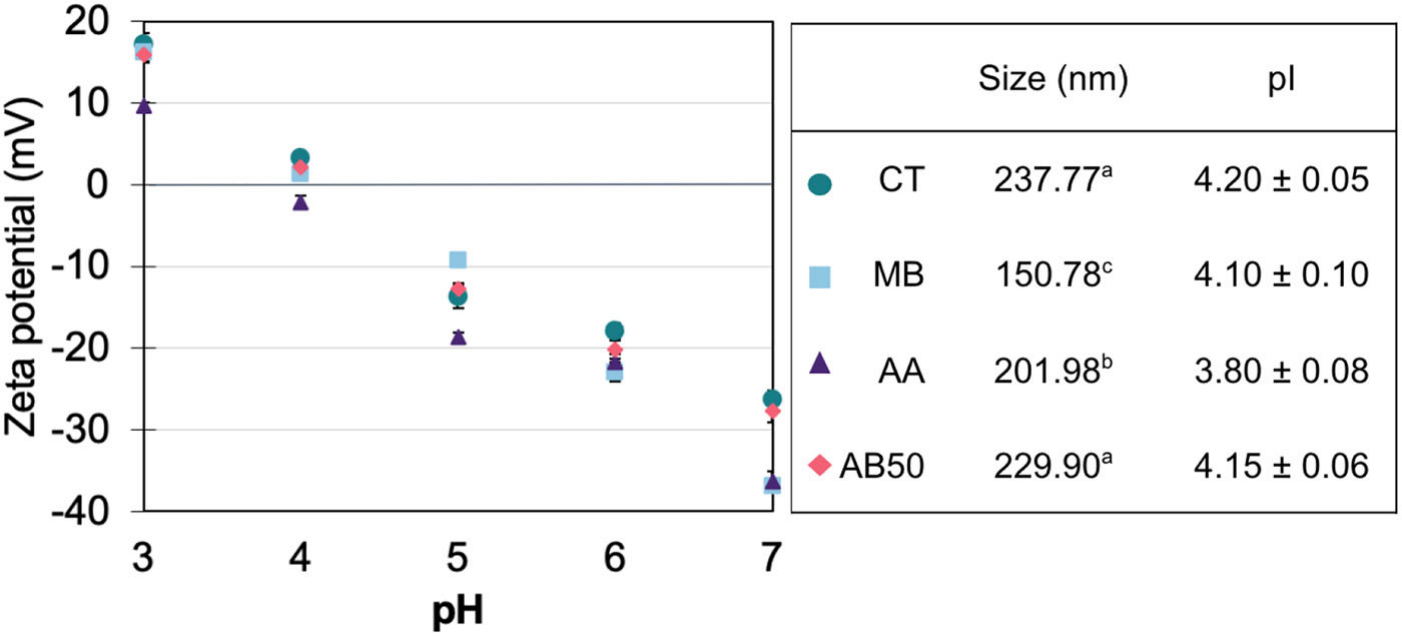
Zeta potential (left), hydrodynamic diameter (at pH 7.0), and isoelectric point (pI) of SPP samples (right) treated with sodium metabisulfite (MB), ascorbic acid (AA), blanching plus ascorbic acid (AB50), and native protein (CT). Different letters in the same column indicate significant differences (*p <* 0.05).

Furthermore, a shift from positive to negative zeta potential values was observed between pH 4 and 5, corresponding to the isoelectric points of the proteins (Figure 1‐right). The estimated pI values (3.8 to 4.2) fall within the range commonly reported for sweet potato proteins (pI ∼4.0) (T. H. Mu et al. 2009; Arogundade and Mu 2012). Minor variations among treatments may be associated with differences in phenolic content, since phenolic compounds can interact with protein surface groups and influence surface charge measurements, which may be related to differences in phenolic content as further explored through phenolic quantification.

### 3.2 Chemical Characterization of the Extracts

Protein concentration varied among treatments (Table 1). While CT, MB, and AB50 showed similar values ranging from 36.50 to 38.93%, AA exhibited a substantially lower protein concentration (20.50%), representing an approximately 45% reduction relative to the other samples. This reduction may be associated with the slight shift in the apparent isoelectric point observed for this treatment (pI ∼3.8 – Figure 1), since a mismatch between the precipitation pH and the isoelectric point can reduce precipitation efficiency by increasing solubility.

**TABLE 1:** Protein content, phenolic compounds, and antioxidant activity of the samples under different enzymatic inactivation treatments by inorganic compound (sodium metabisulfite—MB), organic compound (ascorbic acid—AA), blanching with ascorbic acid (AB50), and control (CT).

Sample	Protein (% w/w)	Total phenolics (mg GAE/g)	Antioxidant activity (uM Trolox/L)
CT	38.93 ± 2.21^a^	9.96 ± 0.43^a^	565.32 ± 9.26^d^
MB	36.50 ± 1.55^a^	8.82 ± 0.19^b^	895.04 ± 1.55^a^
AA	20.50 ± 0.28^b^	6.40 ± 0.20^c^	613.21 ± 14.61^c^
AB50	38.36 ± 2.90^a^	9.50 ± 0.34^a^	866.16 ± 9.42^b^

Different letters in the same column indicate significant differences (*p* < 0.05).

Phenolic compounds are recognized constituents of sweet potato matrices (de Albuquerque et al. 2019) and are known to interact with proteins through hydrogen bonding, electrostatic, and hydrophobic interactions (Arogundade and Mu 2012). Hydrophobic interactions may be particularly relevant, as the aromatic rings of phenolic compounds can associate with hydrophobic amino acid residues in sweet potato proteins, such as alanine, isoleucine, leucine, phenylalanine, tryptophan, and valine (Feng et al. 2023; T. H. Mu et al. 2009). Since these interactions can lead to co‐precipitation during isoelectric recovery, total phenolic content was quantified (Table 1).

In general, all samples showed higher phenolic concentration than the 3.76 mg GAE/g reported by Arogundade and Mu (2012), following the order CT > AB50> MB > AA. The highest phenolic concentrations were observed in CT and AB50 (9.96 and 9.50 mg GAE/g, respectively), with no significant difference between them. A similar trend was observed for protein concentration and phenolic content, with protein‐rich extracts generally exhibiting higher phenolic levels. The elevated phenolic content in AB50 may be partly related to thermal treatment, which can enhance phenolic release by increasing solubility and disrupting cellular structures (Antony and Farid 2022). Heat‐induced protein unfolding may additionally favor phenolic–protein associations by exposing hydrophobic regions (Shahidi and Dissanayaka 2023).

The AA sample showed both the lowest protein concentration and the lowest phenolic content (6.40 mg GAE/g), which may reflect reduced co‐precipitation. In contrast, the higher phenolic content observed in the control sample indicates that a substantial fraction of phenolic compounds remained associated with the recovered protein fraction, despite the presence of PPO activity (Shahidi and Dissanayaka 2023).

### 3.3 Fourier‐Transform Infrared (FTIR)

Given the differences in protein concentration and phenolic content among the extracts, FTIR analysis was performed to provide additional information regarding their molecular structure and functional groups (Figure 2). The Amide I band (1600–1700 cm^−^^1^), primarily associated with C = O stretching vibrations of peptide bonds, is characteristic of proteins and was observed in all extracts, with a prominent absorption around 1650 cm^−^^1^ (Figure 2), confirming the proteinaceous nature of the samples (Czubinski and Dwiecki 2017; Yan et al. 2023).

**FIGURE 2:**
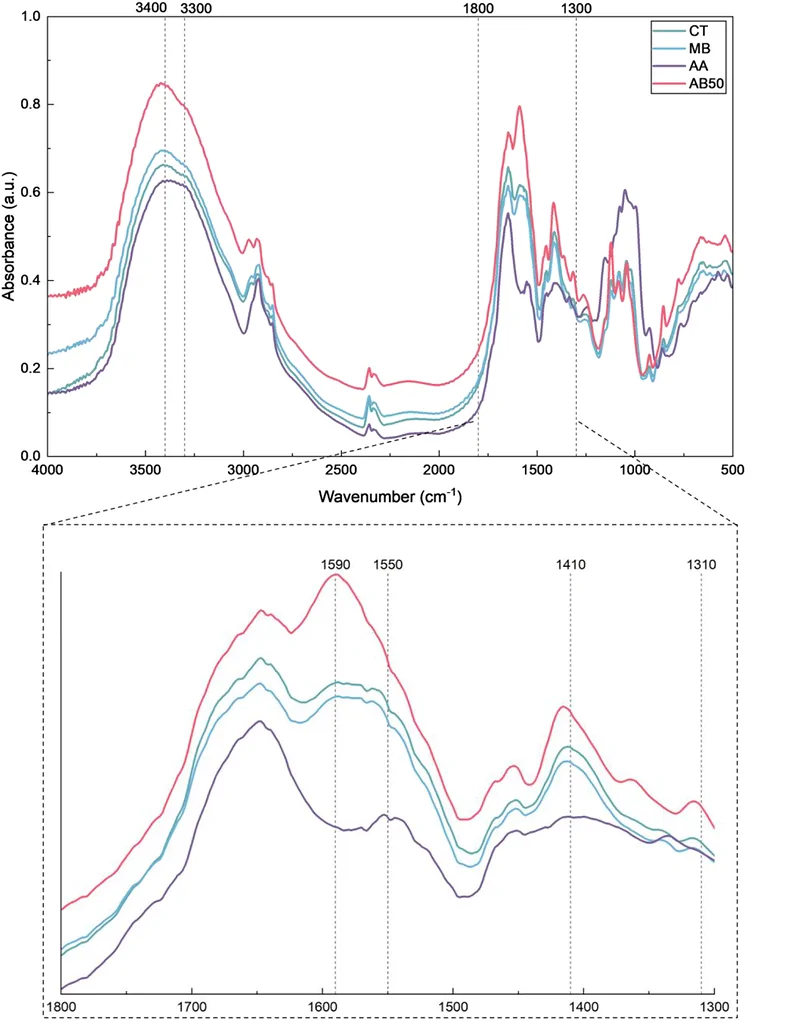
Fourier‐transform infrared (FTIR) spectra of sweet potato protein extracts: control (CT), treated with sodium metabisulfite (MB), ascorbic acid (AA), and ascorbic acid at 50°C (AB50). The spectra were recorded in the range of 400–4000 cm^−^^1^. The enlarged panel highlights the spectral region between 1800 and 1300 cm^−^^1^, including the characteristic protein bands (amide I and amide II). Vertical dashed lines indicate the characteristic wavenumbers discussed in the text.

The Amide II region (1500–1600 cm^−^^1^), mainly attributed to C–N stretching and N–H bending vibrations (Yan et al. 2023), also showed noticeable differences among treatments. CT, MB, and AB50 exhibited bands centered at approximately 1590 cm^−^^1^, whereas AA exhibited a shift toward lower wavenumbers (∼1550 cm^−^^1^). Since this region is highly sensitive to the molecular environment of peptide groups, the observed displacement suggests treatment‐induced structural rearrangements and differences in protein–matrix interactions. The distinct spectral profile of AA may be associated with its lower protein and phenolic contents relative to the other extracts.

Additional differences were observed in the Amide A region (3300–3400 cm^−^^1^), which is associated with N–H stretching vibrations and hydrogen bonding (Yan et al. 2023). This spectral region may also be influenced by hydroxyl‐containing compounds, including phenolics, due to overlap between N–H and O–H vibrations. AA exhibited a broader and less defined band profile, whereas AB50 showed a more pronounced absorption pattern, while CT and MB presented intermediate behavior. These variations suggest differences in hydrogen‐bonding interactions and molecular organization among the extracts.

Phenolic compounds may occur in protein extracts either as free molecules or associated with proteins through non‐covalent interactions such as hydrogen bonding and hydrophobic interactions. In addition to the Amide A region, bands between 1410 and 1310 cm^−^^1^, commonly associated with phenolic structures (Malik et al. 2016), also differed among treatments. CT, MB, and AB50 exhibited more pronounced absorptions in this region, whereas AA showed reduced intensity. Interestingly, this behavior followed the same trend observed for total phenolic content, supporting the contribution of phenolic compounds to the spectral profile of the extracts. Considered in conjunction, the FTIR results suggest that the anti‐browning treatments modified the molecular organization of the protein extracts and are consistent with the presence of phenolic‐associated functional groups and may reflect non‐covalent protein–phenolic associations.

### 3.4 Thermal Properties

The thermal transitions of native and treated sweet potato protein extracts are presented in Figure 3A. The denaturation peak temperature (*Td*), a key indicator of protein thermal stability, varied among treatments, indicating structural modifications. The MB sample exhibited a slightly higher *Td* than CT (82°C *vs*. 80°C), indicating that a higher temperature was required to initiate protein denaturation. However, this sample also presented the lowest enthalpy change (*ΔH* = 0.15 J/g), indicating that less energy was required to complete the denaturation process. The reduced *ΔH* may reflect disruption of stabilizing interactions within the protein structure, possibly associated with the reducing action of sodium metabisulfite on disulfide bonds (N. Li et al. 2012), which can promote partial unfolding (Z. K. Zhao et al. 2019).

**FIGURE 3:**
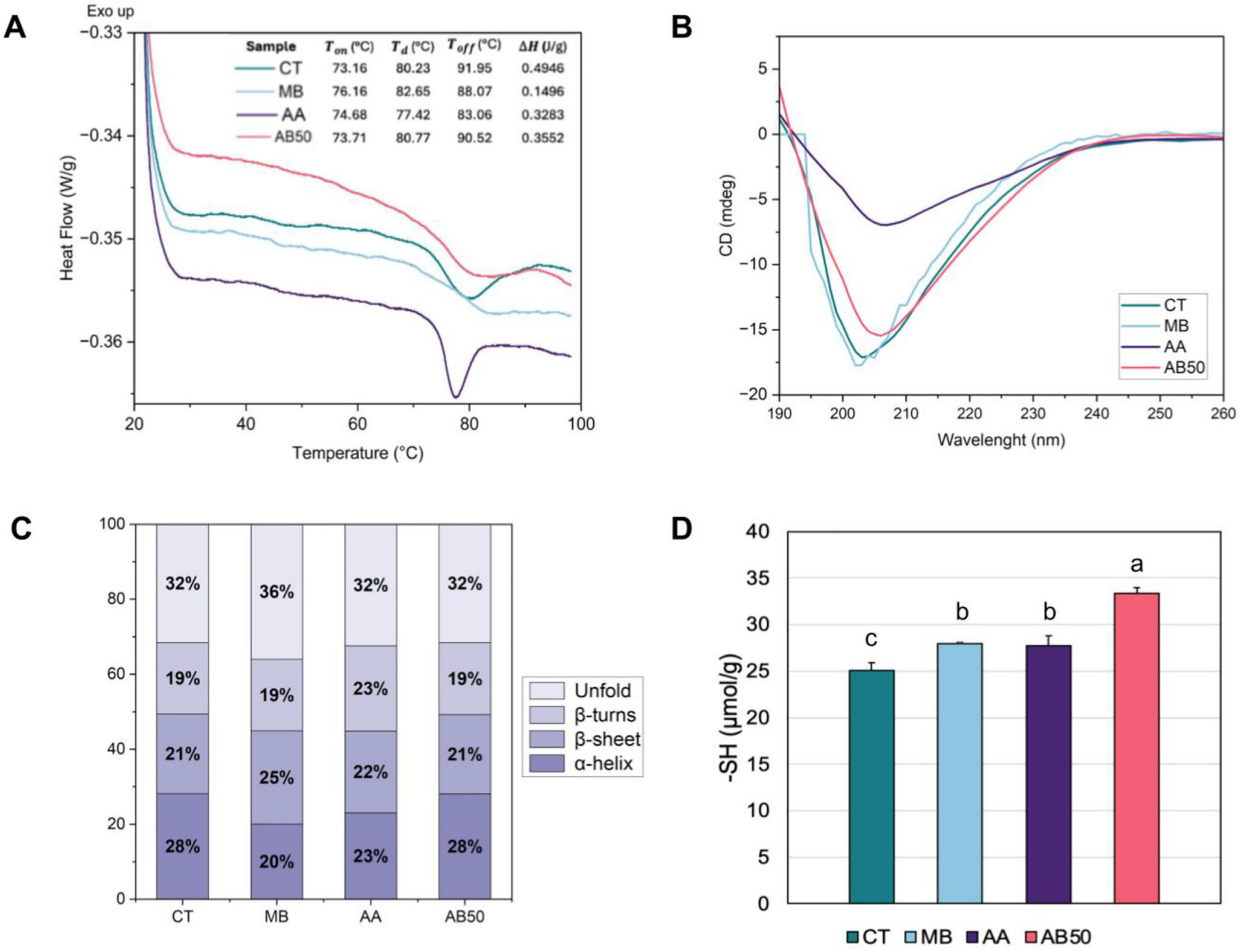
Structural characterization of sweet potato protein extracts: control (CT), treated with sodium metabisulfite (MB), ascorbic acid (AA), and ascorbic acid at 50°C (AB50). (A) Differential scanning calorimetry (DSC) thermograms showing onset temperature (*Ton*), peak denaturation temperature (*Td*), endset temperature (*Toff*), and denaturation enthalpy (*ΔH*). (B) Far‐UV circular dichroism (CD) spectra. (C) Estimated secondary structure composition (%) derived from CD spectra. (D) Free sulfhydryl (–SH) group content. Bars represent the mean ± standard deviation. Different lowercase letters above the bars indicate significant differences among treatments (*p* < 0.05).

The AA sample also exhibited reduced thermal stability, with lower *Td* and *ΔH* (0.33 J/g) compared to CT (0.49 J/g). This may be related to structural rearrangements or partial unfolding induced by ascorbic acid during extraction. Additionally, the lower protein and phenolic contents observed in AA may have limited the formation of protein–phenolic complexes, which are reported to contribute to thermal stabilization through covalent and non‐covalent interactions (Wang et al. 2024). In contrast, AB50 presented a *ΔH* of 0.35 J/g, suggesting partial modification of the protein structure, as later supported by the circular dichroism results, likely resulting from the moderate heating step that promoted limited unfolding while still preserving stabilizing interactions within the protein matrix.

Onset (*Ton*) and endset (*Toff*) denaturation temperatures followed trends consistent with *Td*. CT and AB50 showed broader transition ranges, whereas AA exhibited a narrower range. The *Toff* values of CT, AB50, and MB (92°C, 91°C, and 88°C, respectively) were within the range reported for sweet potato proteins (Arogundade et al. 2021), while AA presented a lower *Toff* (83.06°C), reinforcing its reduced thermal stability.

### 3.5 Secondary Structure

The structural changes suggested by the thermal analysis were also reflected in the secondary structure of SPPs, evaluated by circular dichroism (CD) spectroscopy. As shown in Figure 3B, treated samples exhibited slight alterations in secondary structure compared to the control (CT), indicating treatment‐induced conformational changes in the proteins. Protein conformation is highly sensitive to environmental conditions such as pH, temperature, and the presence of ligands. Treatments that promote partial unfolding can disrupt native arrangements, including α‐helices and β‐sheets, leading to exposure of hydrophobic regions and shifts in the relative content of secondary structure elements (Alizadeh Sani et al. 2024).

The AA sample exhibited the lowest CD signal intensity among all treatments. Considering the lower protein concentration in this sample (20.50%), and that far‐UV CD signals arise mainly from peptide bond absorption, the reduced intensity is likely associated with the lower concentration of absorbing chromophores rather than solely structural differences.

The MB sample exhibited a pronounced negative band at 208 nm, typically associated with α‐helix contributions, indicating modifications in the protein secondary structure. Quantitative analysis confirmed that the α‐helix content decreased from 28% in CT to 20.5% and 24.1% in the MB and AA samples, respectively (Figure 3C). In the MB treatment, sodium metabisulfite may influence protein secondary structure through the release of bisulfite ions, which can react with cysteine residues or carbonyl groups, disrupting disulfide bonds and modifying intramolecular hydrogen bonding. These reactions can lead to reduced α‐helix content and increased β‐sheet or random coil structures (Du et al. 2023). Such structural rearrangements are consistent with the lower thermal stability and enthalpy observed by DSC for this treatment (Figure 3A), suggesting partial unfolding and destabilization of the protein structure.

In the case of the AA sample, the reduction in α‐helix content may be associated with interactions between ascorbic acid and amino acid side chains, which may disrupt hydrogen bonding and induce secondary‐structure rearrangements, resulting in decreased α‐helix regions and increased β‐sheet content. Similar structural effects have been reported in food proteins subjected to sulfite treatments (Du et al. 2023).

The concurrent decrease in α‐helix and increase in β‐sheet content in AA and MB are characteristic of partial unfolding and structural reorganization, consistently with the DSC results. This structural transition may also reflect the formation of protein–phenolic complexes, whose association with proteins depends on structural compatibility and molecular characteristics (Castanha et al. 2023; Sęczyk et al. 2019).

In contrast, CT and AB50 exhibited similar secondary‐structure compositions (Figure 3C), suggesting that the thermal step may mitigate part of the structural disruption observed for ascorbic acid alone. Because α‐helices represent a more ordered and compact conformation, their relative preservation is consistent with greater structural stability, whereas reductions in α‐helical content are commonly associated with increased conformational flexibility (Günal‐Köroğlu et al. 2023).

### 3.6 Protein Oxidation

As shown in Figure 3D, all treated samples exhibited higher free sulfhydryl (–SH) contents (27.98, 27.74, and 33.34 µmol/g for MB, AA, and AB50, respectively) than the control (25 µmol/g), with statistically significant differences. Free –SH groups are indicators of oxidative modifications and associated structural changes in proteins (Wu et al. 2024), and their higher concentrations are generally associated with lower levels of oxidative damage (R. Liu et al. 2024). This pattern is consistent with the controlled PPO inactivation applied prior to the treatments, which likely limited quinone‐mediated oxidation.

In the AA sample (27.74 µmol/g), the slight increase in free –SH groups may also be related to the antioxidant properties of ascorbic acid, which can act as a reducing agent and metal chelator. Structural changes induced by ascorbic acid, such as increased flexibility and partial unfolding, may have contributed to the exposure of sulfhydryl groups that remained available in the system. A similar trend was observed for the MB treatment. Sodium metabisulfite can generate sulfite ions with reducing capacity and may cleave disulfide bonds (Du et al. 2023), which can contribute to maintaining higher –SH availability. These effects likely act in combination with the previously controlled PPO activity.

The sample AB50 showed the highest concentration of free sulfhydryl groups. Although no marked changes in secondary structure were observed for this sample, DSC results showed modifications in thermal behavior, with a lower denaturation enthalpy compared to the control (Figure 3A), suggesting alterations at tertiary and quaternary structural levels. Such conformational adjustments may favor the exposure of internal –SH groups. This sample also presented one of the highest phenolic contents, and phenolic compounds can protect proteins from oxidative damage through radical scavenging, metal chelation, and protein–phenolic interactions that limit protein carbonylation and structural destabilization (Feng et al. 2023).

### 3.7 Functional Properties

#### 3.7.1 Solubility

All samples exhibited the typical U‐shaped solubility profile as a function of pH (Figure 4), with minimum solubility near the isoelectric point (∼pH 4). Under these conditions, proteins carry minimal net charge, which reduces electrostatic repulsion and favors protein–protein interactions, leading to lower solubility (H.‐X. Zhou and Pang 2018). This behavior closely paralleled the zeta potential results (Figure 1), in which minimal surface charge corresponded to reduced solubility, highlighting the close relationship between protein surface charge and solubility, both of which are governed by intrinsic and extrinsic structural characteristics (Gao et al. 2024).

**FIGURE 4:**
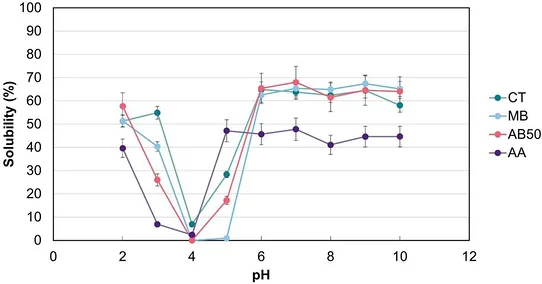
Variation of the solubility of sweet potato protein extracts according to pH: control (CT), AB50 (treated with 0.80 mM ascorbic acid solution at 50°C), AA (treated with 0.80 mM ascorbic acid), and MB (treated with 0.05 mM sodium metabisulfite). Bars represent the mean ± standard deviation.

Although all samples followed this general trend, differences in the curvature of the solubility profiles were evident. The control sample exhibited a sharper and narrower solubility minimum, whereas all treated samples showed broader and less pronounced minima. This pattern indicates that treated proteins were less prone to aggregation near the isoelectric region and maintained greater protein–solvent interactions across a wider pH range.

MB and AB50 showed solubility profiles comparable to the control, but AB50 reached the highest solubility (∼68% at pH 7). DSC results showed a lower denaturation enthalpy for AB50 compared to CT (Figure 3A), suggesting modifications in higher‐order structural interactions. Such conformational adjustments may favor exposure of hydrophilic regions and enhance protein–solvent interactions, contributing to higher solubility. Protein solubility reflects the balance between protein–protein and protein–solvent interactions and is highly pH‐dependent. Molecular flexibility and amino acid composition also contribute to this behavior. Storage proteins rich in glutamic (Glu) and aspartic acid (Asp) residues tend to exhibit higher solubility, and these amino acids are abundant in sweet potato proteins (T. H. Mu et al. 2009), resulting in higher solubility than other vegetable sources such as pea (Sajib et al. 2023) and rice proteins (D. Li et al. 2023).

For the AA sample, the shift in isoelectric point to pH 3.8 (Figure 1) likely influenced its solubility profile, which showed the lowest overall values, reaching a maximum of ∼48% at pH 5. This behavior may be associated with treatment‐induced conformational changes and altered charge distribution, which modify protein–protein and protein–solvent interactions.

#### 3.7.2 Emulsifying Capacity and Stability

As shown in Figure 5A, treated samples exhibited higher emulsifying capacity than the control, along with improved emulsion stability after 24 h (Figure 5B). Since the emulsifying properties of plant proteins depend on molecular features such as amino acid composition, surface hydrophobicity, and chain flexibility (Kaczmarek et al. 2025), these improvements suggest that the treatments promoted structural modifications that enhanced interfacial activity. Partial unfolding can increase molecular flexibility and expose hydrophobic regions, facilitating protein adsorption and rearrangement at the oil–water interface (R. Zhang et al. 2023). The control sample presented an EAI of 7.56 m^2^/g, which falls within the range reported for other root tuber proteins, such as potato protein (4.5 m^2^/g; Schmidt et al. 2018) and yam protein (∼10 m^2^/g; Tang et al. 2020).

**FIGURE 5:**
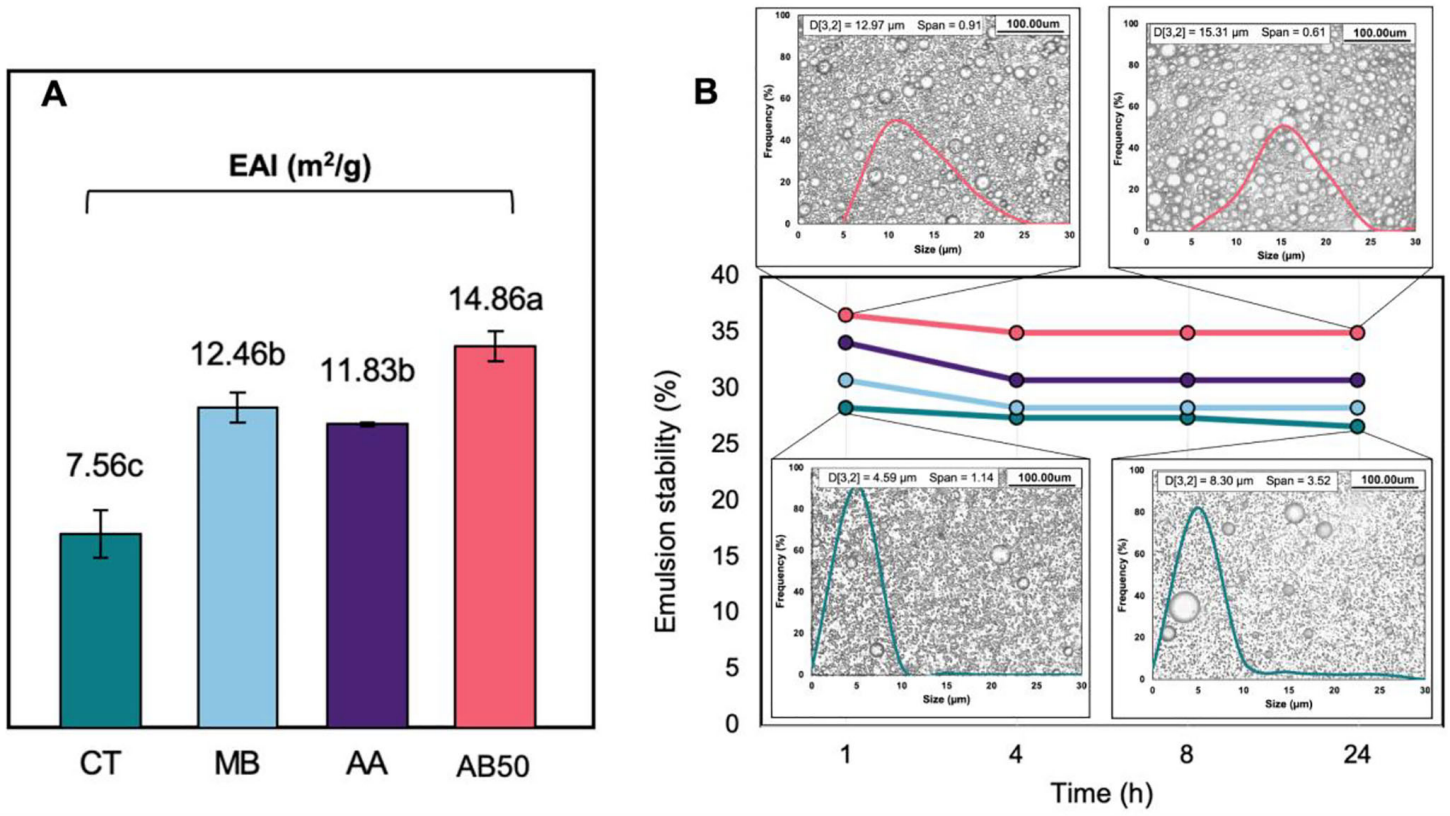
(A) Emulsifying activity index (EAI) of sweet potato protein extracts: control (CT), sodium metabisulfite (MB), ascorbic acid (AA), and ascorbic acid at 50°C (AB50). Different lowercase letters indicate significant differences among treatments (*p* < 0.05). (B) Emulsion stability during 24 h of storage at 25°C, representative optical micrographs of emulsions prepared with 1% (w/w) protein extract as the aqueous phase, and quantitative droplet size analysis, including D[3,2] and Span. Scale bar = 100 µm.

The AA sample showed enhanced emulsifying capacity (11.83 vs 7.56 m^2^/g, respectively) and greater emulsion stability compared to the control. This behavior may be associated with interactions between ascorbic acid and SPPs that improve protein adsorption at the oil–water interface (Fazelioskouei et al. 2023). In addition, treatment‐induced structural modifications (Figure 3B–C) may have contributed to these results. A reduction in α‐helix content is often related to increased molecular flexibility, which can facilitate adsorption at the oil–water interface, promote interfacial rearrangement, and improve emulsion stability (Günal‐Köroğlu et al. 2023).

The best emulsifying performance was observed for AB50. This behavior may be associated with modifications in higher‐order protein structure, as suggested by DSC results (Figure 3A), which indicated changes in denaturation enthalpy and possible alterations in tertiary or quaternary interactions. Such modifications can increase exposure of hydrophobic and reactive groups (Figure 3D), improving interfacial adsorption. In addition, AB50 presented the highest phenolic content and free sulfhydryl levels, which may favor protein–phenolic interactions and contribute to emulsion stability (Santos et al. 2022).

Figure 5B presents optical micrographs of emulsions prepared with CT and AB50 immediately after preparation and after 24 h. Quantitative droplet analysis supported the visual observations. AB50 showed only a slight increase in D[3,2] (12.97 to 15.31 µm), while Span decreased from 0.91 to 0.61, indicating a stable and narrower droplet size distribution over storage. In contrast, CT exhibited an approximately twofold increase in D[3,2], accompanied by a marked increase in span from 1.14 to 3.52, indicating droplet growth and a broader size distribution consistent with coalescence. Considered in conjunction, these results indicate that the AB50 emulsion exhibited greater structural stability during storage, characterized by a narrower droplet size distribution and reduced coalescence, whereas the CT sample underwent progressive droplet growth and coalescence, as shown by the micrographs and quantitative droplet analysis.

These findings are consistent with the zeta potential results (Figure 1), in which the control exhibited lower net surface charge, reducing electrostatic repulsion between droplets and favoring aggregation. In contrast, treated samples presented higher net zeta potential values, which promote electrostatic stabilization and limit droplet aggregation (Khan et al. 2014). Emulsion stabilization is also influenced by the protein's ability to adsorb and form interfacial films, which depends on molecular interactions and the distribution of hydrophilic and hydrophobic regions.

#### 3.7.3 Antioxidant Activity

In emulsion‐based systems, stability involves not only kinetic aspects, such as droplet aggregation and coalescence, but also chemical stability, since lipid oxidation is a major factor limiting shelf life and quality (Yan et al. 2022). Therefore, ingredients that simultaneously provide interfacial stabilization and antioxidant protection are particularly valuable in food emulsions. In this regard, the amino acid composition of proteins can also contribute to antioxidant behavior. Sweet potato proteins are rich in glutamic (Glu) and aspartic acid (Asp) (T. H. Mu et al. 2009), whose negatively charged side chains can chelate pro‐oxidant metal ions (Wen et al. 2020), potentially helping to limit oxidation reactions in emulsion systems.

In this context, the antioxidant activity of native and treated sweet potato protein extracts was evaluated. As shown in Table 1, the treatments significantly influenced the antioxidant potential of SPPs. The control sample (CT) exhibited the lowest antioxidant activity (565.32 µM Trolox/L). This relatively low value may be associated with the presence of active polyphenol oxidase (PPO), which catalyzes the oxidation of phenolic compounds into quinones, thereby reducing antioxidant capacity (Feng et al. 2023), despite the high total phenolic content measured in this sample (9.96 mg GAE/g). Although antioxidant activity has been reported for proteins from tuberous crops such as potato, sweet potato, and yam (H. Liu et al. 2023; Sung et al. 2025; M. Zhang et al. 2019), these studies predominantly focus on purified proteins, hydrolysates, or bioactive peptides. In contrast, information on crude protein extracts from tuberous roots remains limited.

In the AA sample, the antioxidant capacity was relatively low (613 µM Trolox/L) in comparison to the other treatments, which can be attributed to the structural changes in the protein induced by AA, such as partial unfolding or disruption of α‐helix regions (Figure 2B‐C), which may have exposed reactive sites to aggregation or reduced their accessibility, limiting the exposure of antioxidant‐active amino acids (Luo et al. 2024). Although ascorbic acid is recognized for its antioxidant properties, the AA sample exhibited lower total phenolic and protein contents, which reduced the pool of phenolic compounds available for interaction with proteins, forming fewer complexes that are usually associated with higher antioxidant activity.

The AB50 sample demonstrated a pronounced antioxidant effect (866.16 µM Trolox/L). This behavior may be associated with structural rearrangements reflected by the higher concentration of free sulfhydryl groups, which indicate exposure of previously buried reactive groups. Free sulfhydryl groups are recognized contributors to radical scavenging (Figure 3D) and, together with the higher phenolic and protein contents (Table 1), contribute to the antioxidant activity observed. In addition, the higher solubility of the AB50 sample (Figure 4) likely favored the interaction of these antioxidant components with the reaction medium. The MB sample showed the highest antioxidant activity (895 µM Trolox/L – Table 1) compared to CT (565 µM Trolox/L). MB also showed a significant difference in the content of free sulfhydryl groups (Figure 3D), suggesting that this may also be associated with the increase in antioxidant activity. This result is consistent with its relatively high protein and phenolic contents. In addition, circular dichroism analysis indicated more pronounced structural modifications in this sample (Figure 3 B‐C), suggesting alterations in higher‐order interactions that may increase the exposure of reactive groups involved in radical scavenging (Du et al. 2023). Sodium metabisulfite is also recognized for its antioxidant and preservative properties, which may have contributed to the overall antioxidant response.

## 4 Conclusion

This study demonstrated that targeted processing strategies based on controlled enzymatic inactivation effectively enhanced the structural and functional properties of proteins from sweet potato by‐products, yielding extracts with improved emulsifying and antioxidant functionality. Despite presenting the lowest protein concentration, the AA treatment maintained relevant emulsifying activity and stability, suggesting that moderate structural modifications can still promote interfacial functionality even when compositional parameters are reduced. The control sample showed lower antioxidant performance and emulsion stability, confirming that polyphenol oxidase activity negatively affects protein functionality. The combined use of ascorbic acid and heat (AB50) was more effective than individual treatments, providing the best balance between emulsifying performance and antioxidant activity. FTIR analyses suggested that protein–phenolic interactions contributed to the structural rearrangements associated with these functional improvements. Although sodium metabisulfite (MB) resulted in the highest measured antioxidant activity, AB50 achieved comparable antioxidant and phenolic levels while surpassing MB in sulfhydryl exposure and emulsifying performance. These findings indicate that AB50 can provide similar or superior functionality compared with inorganic antibrowning agents while avoiding the use of sulfite‐based additives, making it a promising clean‐label alternative for protein ingredient production. Overall, these findings demonstrate that sweet potato protein extracts can be functionally tailored through mild and targeted processing. This strategy adds value to sweet potato by‐products and supports their application as multifunctional, clean‐label ingredients in food formulations without relying on synthetic additives associated with environmental or health concerns.

## Author Contributions

**Isabella de Almeida Pina**: data curation, formal analysis, investigation, methodology, writing – original draft, visualization, validation. **Ana Clara Troya Raineri Fiocco**: conceptualization, data curation, investigation, methodology, writing – original draft, validation, visualization. **Isabela Simões Soares**: formal analysis, investigation, methodology, writing – original draft, visualization. **Carolina Siqueira Franco Picone**: conceptualization, resources, writing – review and editing, supervision, visualization, project administration, funding acquisition.

## Conflicts of Interest

The authors declare no conflicts of interests.

## Data Availability

Data will be made available on request.
